# Case of Immune Checkpoint Inhibitor Induced Myasthenia Gravis

**DOI:** 10.7759/cureus.58651

**Published:** 2024-04-20

**Authors:** Manoja Gullapalli, Narenraj Arulprakash, Mazin Safar, Emily Kocurek

**Affiliations:** 1 Internal Medicine - Pediatrics, University of Arkansas for Medical Sciences, Little Rock, USA; 2 Vascular Neurology, Johns Hopkins University School of Medicine, Baltimore, USA; 3 Hematology and Medical Oncology, University of Arkansas for Medical Sciences, Little Rock, USA; 4 Medicine, Division of Pulmonary and Critical Care, University of Arkansas for Medical Sciences, Little Rock, USA

**Keywords:** cardiotoxicity, aseptic meningitis, neurotoxicity, myasthenia gravis, immune checkpoint inhibitors

## Abstract

An 85-year-old man was diagnosed with hepatocellular carcinoma (HCC) and was initially treated with transarterial chemoembolization (TACE) and sorafenib. He was then switched to nivolumab and ipilimumab in view of sorafenib intolerance and disease progression. Subsequently, he developed dysphagia and generalized dyspnea culminating in hypercapnic respiratory failure requiring intubation. After an extensive workup, the etiology of his fluctuating respiratory issues was narrowed down to a likely neuromuscular process. Although antibodies to acetylcholine receptors (anti-AChR Ab) were negative, he was treated with high-dose steroids due to clinical concern for Immune Checkpoint Inhibitor (ICI) neurotoxicity. His recovery post immune suppression and absence of recurrence after ICI cessation suggested the possibility of this being an ICI neurotoxicity manifesting with myasthenic symptoms. Incidentally, he also had evidence of aseptic meningitis on cerebrospinal fluid analysis further strengthening this diagnosis. This case illustrates the importance of early recognition of ICI toxicity which will in turn lead to initiating treatments sooner and also decreasing the length of illness.

## Introduction

Immune checkpoints are a normal part of the immune system and function to prevent immune response against normal healthy cells via inhibiting T cell responses. Cancer cells can use immune checkpoints to evade detection, and monoclonal antibodies that block immune checkpoints are a breakthrough in cancer therapeutics. Programmed death-1 receptor (PD-1), ligand (PDL-1), and cytotoxic T-lymphocyte antigen-4 (CTLA-4) are relatively well studied. T-cell modulators, CTLA4 blockade antibodies (ex: ipilimumab), and anti-PD1/PDL1 (ex: nivolumab) are some of the most widely used anti-tumor ICI agents [1-4]. The increased use of ICIs in the last decade has revealed the potential for systemic side effects, including severe adverse effects on the nervous system, and myasthenia gravis is a rare presentation of ICI neurotoxicity [5-8]. ICI-related Myasthenia Gravis (MG) has been reported to be the most common neuromuscular immune-related adverse event, and ICI-related MG appears to cause respiratory weakness more frequently than classical MG [7]. With ICI-related immune complications, cases have been reported to be associated with autoimmune conditions, and presentation is different compared to the typical presentation of autoimmune conditions. With high clinical suspicion, the diagnosis could be made quickly, leading to prompt treatment.

## Case presentation

**Figure 1:**
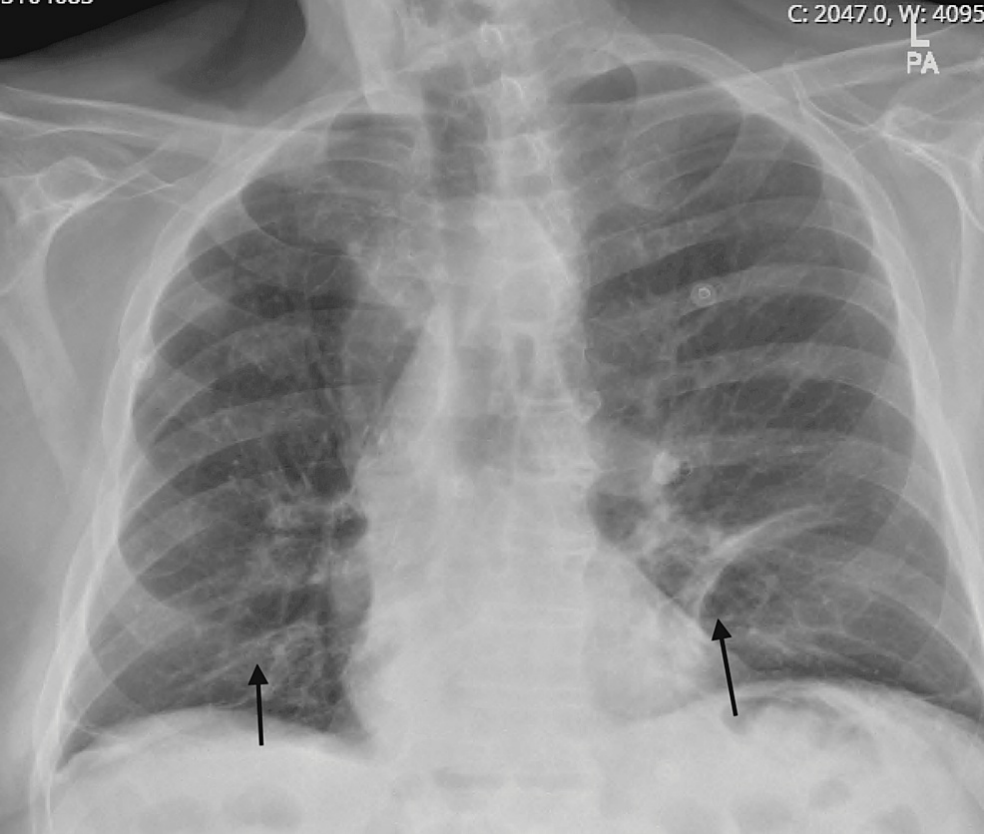
Chest x-ray with subsegmental atelectasis in the lung bases in the setting of acute respiratory failure.

An 85-year-old Caucasian male with hepatocellular carcinoma was initially treated with transarterial chemoembolization (TACE) and sorafenib. He subsequently received 19 cycles of nivolumab after sorafenib intolerance and disease progression. Two months before the presentation, new lung metastases were detected, and he was started on ipilimumab. After receiving his fourth cycle of nivolumab/ipilimumab, he began to have dysphagia and weakness and eventually presented to the hospital with shortness of breath. He was found to be in respiratory failure and started on bilevel-positive airway pressure (BiPAP). Chest x-ray showed atelectatic changes (Figure 1). He was treated for chronic obstructive pulmonary disease (COPD) exacerbation with steroids and antibiotics. His respiratory distress resolved, and he was discharged home within two days. Four days later, he was re-admitted with worsening dyspnea. Shortly, he developed shock and bradycardia with electrocardiography (EKG) showing junctional bradycardia. His troponin was elevated, but his echocardiogram showed a grossly normal ejection fraction. An ICI cardiotoxicity was suspected, and a myocardial perfusion scan was performed which was positive for reversible perfusion defect, but an MRI could not be performed and a biopsy was not pursued [5,6]. His sputum culture showed Pseudomonas aeruginosa. His shock required vasopressor support and pulse dose steroids. On day 4 of hospitalization, he developed respiratory failure and altered mental status requiring emergent intubation. His antibiotic coverage was broadened, and he was given stress-dose steroids. He was extubated but subsequently developed hypercapnic respiratory failure requiring re-intubation. A neurologist was consulted given recurrent episodes of respiratory failure with concern for possible neuromuscular junction disorder causing the weakness. On neurological assessment, the clinical picture was consistent with myasthenia gravis (MG) as evidenced by dysphagia and episodic weakness which included respiratory muscles. The rest of the neurological exam was normal, but his negative inspiratory force (NIF) and vital capacity (VC) were low (Table 1). CSF findings were concerning for aseptic meningitis (Table 2). Myasthenia antibodies resulted in negative as well. Inflammatory myopathy (anti-Jo 1) and AIDP (anti-GM1 Abs). Due to clinical concern for ICI neurotoxicity, methylprednisolone was initiated (1 g intravenously daily for five days) [2,7]. Ultimately the clinical picture was thought to be most consistent with seronegative MG [7,9]. Since the patient exhibited fluctuating weakness, other causes including inflammatory myopathy and acute inflammatory demyelinating polyradiculoneuropathy were considered less likely. The patient showed remarkable recovery in his respiratory function and dysphagia with steroids, and his NIF and VC improved as well (Table 1). He was successfully extubated after four days of steroid treatment and transferred to the floor. He was stable for the remainder of his stay and, at follow up, he reported no further issues with swallowing or respiration. Since he was asymptomatic at a follow-up clinic appointment off of any medications, the plan was to follow him clinically without starting long-term immunosuppression.

**Table 1:** Negative inspiratory force (NIF) and vital capacity (VC) in Liters before and after treatment

	Pre-treatment	Post-treatment
NIF	-49	-38
VC	1.78	3.48

**Table 2:** CSF findings concerning aseptic meningitis with 0 RBC and 25 WBC

CSF lab	Value
Protein mg/dL	73
CSF Glucose mg/dL	86
WBCs/$\mu$L	25
RBCs/$\mu$L	0
Gram Stain	Negative
CSF culture	Negative

## Discussion

ICI neurotoxicity is known to cause an array of symptoms involving the brain, spinal cord, nerves, and neuromuscular junction. Manifestations include aseptic meningitis, autoimmune encephalitis, Guillain-Barre syndrome, MG and inflammatory myopathy among others [1-4]. The diagnosis of neurological complications associated with ICIs is usually challenging as these disorders are usually a diagnosis of exclusion. The incidence of myasthenia gravis due to ICI neurotoxicity is estimated to be approximately 0.1%-0.2% [1]. ICI-MG tends to occur in men with a median age of 70. Symptoms are most commonly noted two to 12 weeks after ICI initiation [1]. The anti-PD-1 treatment appears to cause myasthenia gravis more often than other ICIs. Patients often present with fluctuating muscular weakness involving bulbar, ocular, and even respiratory muscles, sometimes associated with axial and proximal limb weakness and/or severe dyspnea. The coexistence of myasthenia gravis and myositis frequently occurs, and this coexistence is a risk factor for the myasthenic crisis that requires intensive care [6,7]. Corticosteroids are generally used as a first therapy when other possible causes are eliminated. Patients may develop immune-related toxicities simultaneously in other organ systems. For example, cases of ICI-related MG coinciding with myositis or myocarditis have been described [5,6]. Anti-acetylcholinesterase receptor or anti-striated muscle antibodies were positive in only about 66% of ICI-related MG cases, and 60% of patients demonstrated electrodiagnostic evidence of MG or myositis [7].

## Conclusions

Seronegativity is reported to be higher in ICI-related MG than in classic MG. Practitioners should monitor for autoimmune adverse effects associated with ICIs closely since presentations could be atypical. Gathering and describing the clinical and serologic characteristics of a larger cohort of patients with ICI-related MG would be beneficial in order to clarify the unique features of this ICI neurotoxicity and potentially facilitate earlier diagnosis. Efforts to store sera of afflicted patients should be encouraged to enable future research focused on the development of more precise diagnostic testing and targeted therapy.
